# Two independent proteomic approaches provide a comprehensive analysis of the synovial fluid proteome response to Autologous Chondrocyte Implantation

**DOI:** 10.1186/s13075-018-1573-4

**Published:** 2018-05-02

**Authors:** Charlotte H. Hulme, Emma L. Wilson, Heidi R. Fuller, Sally Roberts, James B. Richardson, Pete Gallacher, Mandy J. Peffers, Sally L. Shirran, Catherine H. Botting, Karina T. Wright

**Affiliations:** 1 Institute of Science and Technology in Medicine, Keele University, Keele, Staffordshire ST5 5BG UK; 2 Robert Jones and Agnes Hunt Orthopaedic Hospital, Oswestry, Shropshire SY10 7AG UK; 3 Chester Medical School, Chester University, Chester, CH1 4BJ UK; 4 Institute of Ageing and Chronic Disease, University of Liverpool, Liverpool, L7 8TX UK; 5 BSRC Mass Spectrometry and Proteomics Facility, University of St Andrews, North Haugh, Fife, KY16 9ST UK

**Keywords:** Autologous chondrocyte implantation (ACI), iTRAQ proteomics, Label-free quantitation proteomics, Synovial fluid, Cartilage repair, Complement C1S subcomponent, Matrix metalloproteinase 3, MMP3

## Abstract

**Background:**

Autologous chondrocyte implantation (ACI) has a failure rate of approximately 20%, but it is yet to be fully understood why. Biomarkers are needed that can pre-operatively predict in which patients it is likely to fail, so that alternative or individualised therapies can be offered. We previously used label-free quantitation (LF) with a dynamic range compression proteomic approach to assess the synovial fluid (SF) of ACI responders and non-responders. However, we were able to identify only a few differentially abundant proteins at baseline. In the present study, we built upon these previous findings by assessing higher-abundance proteins within this SF, providing a more global proteomic analysis on the basis of which more of the biology underlying ACI success or failure can be understood.

**Methods:**

Isobaric tagging for relative and absolute quantitation (iTRAQ) proteomic analysis was used to assess SF from ACI responders (mean Lysholm improvement of 33; *n* = 14) and non-responders (mean Lysholm decrease of 14; *n* = 13) at the two stages of surgery (cartilage harvest and chondrocyte implantation). Differentially abundant proteins in iTRAQ and combined iTRAQ and LF datasets were investigated using pathway and network analyses.

**Results:**

iTRAQ proteomic analysis confirmed our previous finding that there is a marked proteomic shift in response to cartilage harvest (70 and 54 proteins demonstrating ≥ 2.0-fold change and *p* < 0.05 between stages I and II in responders and non-responders, respectively). Further, it highlighted 28 proteins that were differentially abundant between responders and non-responders to ACI, which were not found in the LF study, 16 of which were altered at baseline. The differential expression of two proteins (complement C1s subcomponent and matrix metalloproteinase 3) was confirmed biochemically. Combination of the iTRAQ and LF proteomic datasets generated in-depth SF proteome information that was used to generate interactome networks representing ACI success or failure. Functional pathways that are dysregulated in ACI non-responders were identified, including acute-phase response signalling.

**Conclusions:**

Several candidate biomarkers for baseline prediction of ACI outcome were identified. A holistic overview of the SF proteome in responders and non-responders to ACI  has been profiled, providing a better understanding of the biological pathways underlying clinical outcome, particularly the differential response to cartilage harvest in non-responders.

**Electronic supplementary material:**

The online version of this article (10.1186/s13075-018-1573-4) contains supplementary material, which is available to authorized users.

## Background

Identification of putative biomarkers that can be used to predict patient outcome prior to treatment for cartilage injury has been highlighted as a key initiative for the prevention of osteoarthritis (OA) by the Osteoarthritis Research Society International [1]. Further, in the United Kingdom, the National Health Service has increased the need to identify accurate prognostic biomarkers for application of the recent National Institute for Health and Care Excellence (NICE) recommendation for use of the cell therapy called *autologous chondrocyte implantation* (ACI) [2].

We recently published the first study [3], to our knowledge, in which a proteomic approach has been used with the aim of identifying candidate biomarkers to predict the success of ACI, a cellular therapy for the treatment of traumatic cartilage injury [4, 5]. This therapy is composed of a two-stage procedure: During the initial surgery (stage I), healthy cartilage is harvested from a minor load-bearing region of the joint, then chondrocytes are isolated and culture is expanded for 3–4 weeks prior to a second surgery (stage II), in which the chondrocytes are implanted into the cartilage defect [5, 6]. Approximately 500 patients have been treated with ACI in our centre, and despite an 81% success rate [7], we have yet to fully understand why some individuals do not respond well. We have identified a biomarker, aggrecanase-1, that, when its activity is undetectable pre-operatively, can be used together with known demographic and injury-associated risk factors to help predict ACI success [8, 9]. However, we have yet to identify a biomarker (or panel of biomarkers) that can be used to accurately predict ACI failure. The identification of such a biomarker(s) for ACI and other cartilage repair strategies would allow for the better stratification of patients prior to joint surgery and may provide candidates for therapies to improve ACI success.

Proteomic analyses remain one of the most widely used methods to identify novel biomarker candidates and have previously been used to identify biomarkers of OA progression (as summarised by Hsueh et al. in 2014 [10]). The synovial fluid (SF) provides an attractive biological fluid for biomarker identification because it bathes the injured joint and therefore contains proteins that might reflect the whole joint environment. Proteomic profiling of the SF, however, is technically difficult owing to the broad dynamic range of proteins present within it [7, 8]. Several unbiased global proteomic studies aimed at the identification of biomarkers within the SF have been completed. Nevertheless, the number of protein ‘hits’ has been somewhat limited, because researchers either have tended to profile SF with no pre-treatment to account for the wide range of proteins [11–16] or have depleted high-abundance proteins [17–22], meaning that the altered quantities of these proteins cannot be considered.

Isobaric tags for absolute and relative quantitation (iTRAQ) is reported to be the most accurate labelling method for quantifying comparative abundance of proteins [23]. When compared with label-free quantitation (LF) proteomics, iTRAQ quantitation has traditionally been considered a more accurate technique [24]; however, as mass spectrometers are improved, these techniques are becoming more comparable, and LF is becoming increasingly popular [25]. Unlike LF proteomics, iTRAQ uses isobaric tags to label the primary amines at the peptide level prior to pooling the samples to enable simultaneous identification and quantitation of the proteins. Fourplex and eightplex labels are available, enabling quantitation of up to eight conditions in a single analysis, thus minimising the number of mass spectrometry runs that can be cost-effective and time-efficient. However, when compared with LF, in which any number of samples can be analysed and compared, iTRAQ labelling limits the number of samples that can be compared, meaning biological replicate samples are often pooled together into relevant biological conditions. iTRAQ proteomics is a commonly used tool for the identification of biomarkers in a plethora of diseases. This proteomic approach has been used to profile the SF proteome [20, 26], successfully identifying differentially abundant protein biomarker candidates for several diseases/conditions.

Our previous study highlighted the potential of using protein equalisation to study low-abundance proteins in human SF, but this identified few differentially abundant proteins in baseline SF, when comparing individuals who did or did not do well following cartilage repair therapy [1]. The aim of the present study therefore was to increase the number of protein biomarker candidates that could be identified for the pre-operative prediction of clinical outcome following ACI and to allow for the assessment of high-abundance proteins that may also strengthen the understanding of the biological processes underlying treatment success.

## Methods

### SF collection and storage

SF was collected as described previously [3, 8, 27] from the knee joints of patients who provided informed consent and following local research ethics committee approval. Immediately prior to both ACI surgeries, stage I (cartilage harvest) and stage II (chondrocyte implantation), 20 ml of saline was injected into the joint and 20 rounds of leg flexion and extension were carried out to allow aspiration of as much SF as possible [3, 27]. SF was then centrifuged at 6000 × *g* for 15 minutes at 4 °C and split into aliquots for long-term storage in liquid nitrogen. The dilution factor of the SF samples was calculated by comparing urea content in SF with matched blood plasma using a QuantiChrom™ Urea Assay Kit (BioAssay Systems, Hayward, CA, USA) according to the manufacturer’s instructions and as described previously [3, 8, 28], and SF samples with a dilution factor > 10 were excluded from the study.

Clinical responders to ACI were defined as individuals who demonstrated a Lysholm score increase of ≥ 10 points at 12 months post-treatment compared with their baseline score, as has been used previously [29–31]. The Lysholm score is a validated [32] patient-self assessment score encompassing knee pain and joint function that ranges from 0 to 100, with 100 representing ‘perfect’ knee function [32, 33]. Thirteen patients were considered as non-responders to ACI, demonstrating a mean decrease in Lysholm score of 14 points (range − 4 to − 46), and 14 SF donors were considered responders with a mean improvement of 33 points (range 17–54).

### Sample preparation and analysis using iTRAQ proteomics (iTRAQ nanoLC-MS/MS)

Total protein was quantified using a Pierce™ 660 nm Protein Assay (Thermo Fisher Scientific, Hemel Hempstead, UK) [34], and a total of 200 μg of SF protein was pooled equally from the donors in each of the following experimental groups: stage I responders (*n* = 8), stage I non-responders (*n* = 7), stage II responders (*n* = 12), and stage II non-responders (*n* = 12) (Table 1). The pooled samples were then precipitated in six volumes of ice-cold acetone overnight at − 20 °C. The precipitates were pelleted by centrifugation at 13,000 × *g* for 10 minutes at 4 °C before being re-suspended in 200 μl of triethylammonium bicarbonate buffer. Eighty-five micrograms of protein for each experimental sample were then subjected to reduction, alkylation (as instructed in the iTRAQ labelling kit; Applied Biosystems, Bleiswijk, The Netherlands). Sequencing Grade Modified Trypsin (10 μg/85 μg of protein; Promega, Madison, WI, USA) was then added to the samples for overnight digestion at 37 °C. Tryptic digests were labelled with the iTRAQ tags according to the manufacturer’s instructions before being pooled into one microcentrifuge tube prior to being dried in a vacuum centrifuge: 114 tag- stage II responders, 115 tag- stage II non-responders, 116 tag- stage I responders and 117 tag- stage I non-responders.

**Table 1:** Demographic data for patient participants whose samples from Stage I or Stage II were analysed who responded clinically (responders) or who did not respond (non-responders) to autologous chondrocyte implantation (ACI)

	Stage I		Stage II		Mann-Whitney *U* test *p* value (A)R v NR- SI; (B) R v NR- SI	Mann-Whitney U test *p* value) (A)SI v SII-R; (B) SI v SII- NR
	Responders (*n* = 8)	Non-responders (*n* = 7)	Responders (*n* = 12)	Non-responders (*n* = 12)		
Difference in Lysholm score	27 (17–38)	− 8 (− 4 to − 17)	34 (17–54)	− 11 (− 4 to − 46)	(A) 0.0003; (B) < 0.0001	(A) 0.21; (B) 0.55
BMI, kg/m^2^	29 (23–31)	27 (24–31)	27 (23–48)	29 (22–36)	(A) 0.94; (B) 0.54	(A) 0.73; (B) 0.68
Age, years	32 (17–49)	40 (25–50)	40 (17–90)	43 (25–52)	(A) 0.28; (B) 0.92	(A) 0.17; (B) 0.58
Male/female sex, *n*	8/0	7/0	11/1	10/2	(A) > 0.99; (B) > 0.99	(A) > 0.99; (B) 0.51
Smoker, *n*	1	2	1	3	(A) 0.54; (B) 0.59	(A) > 0.99; (B) > 0.99
Dilution factor of SF	5 (3–9)	4 (2–7)	4 (1–9)	3 (2–5)	(A) 0.48; (B) 0.25	(A) 0.53; (B) 0.50
Total defect area, cm^2^	14 (0.4–24)	6 (0.6–12)	6 (1–20)	5 (0.6–12)	(A) 0.74; (B) 0.35	(A) 0.45; (B) 0.28
Patella defect, *n*	1	1	4	2	(A) > 0.99; (B) 0.64	(A) 0.60; (B) > 0.99
LFC defect, *n*	2	0	0	0	(A) 0.47; (B) > 0.99	(A) 0.15; (B) > 0.99
LTP defect, *n*	1	0	0	0	(A) > 0.99; (B) > 0.99	(A) 0.15; (B) > 0.99
MFC defect, *n*	2	2	1	6	(A) > 0.99; (B) 0.07	(A) 0.54; (B) 0.63
Trochlea defect, *n*	0	3	2	1	(A) 0.20; (B) > 0.99	(A) 0.49; (B) 0.12
Multiple defects, *n*	1	0	1	1	(A) > 0.99; (B) > 0.99	(A) > 0.99; (B) > 0.99
Unknown defect location, *n*	1	1	4	2	(A) > 0.99; (B) 0.64	(A) 0.60; (B) > 0.99

Footnote: None of the demographic parameters, other than a difference in Lysholm score, showed differences between responders (R) and non-responders (NR) among individuals whose SF from stage I (SI) or stage II (SII) was compared, nor were there differences between individuals who were either responders or non-responders when we compared stage I and stage II samples (*p* ≥ 0.05 by Mann-Whitney *U* test). Data are median (range). *Abbreviations: BMI* Body mass index, *LFC* Lateral femoral condyle, *LTP* Lateral tibial plateau, *MFC* Medial femoral condyle

iTRAQ-labelled peptides were resuspended in 0.6 ml of loading buffer (10 mM monopotassium phosphate [KH_2_PO_4_], 20% acetonitrile [MeCN], pH 3.0), followed by sonication. The pH was adjusted to 3.0 with 0.5 M orthophosphoric acid (H_3_PO_4_). The peptides were separated by strong cation exchange chromatography (SCX) as described previously [35]. A total of 14 SCX fractions were analysed by nano-electrospray ionisation-LC-MS/MS using a TripleTOF 5600 tandem mass spectrometer (AB Sciex, Framingham, MA, USA) as described previously [36].

The raw mass spectrometry data file was subsequently analysed using ProteinPilot 4.5 software with the Paragon™ and ProGroup™ algorithms (AB Sciex) against the human sequences in the UniProtKB/Swiss-Prot database (downloaded in December 2012). Searches were performed using the pre-set iTRAQ settings in ProteinPilot. Trypsin was selected as the cleavage enzyme and methyl methanethiosulphonate for the modification of cysteines with a ‘thorough ID’ search effort. ProteinPilot’s bias correction assumes that most proteins do not change in expression. Finally, detected proteins were reported with a protein threshold [unused ProtScore (confidence)] > 0.05 and used in the quantitative analysis if they were identified with two or more unique peptides with 95% confidence or above. *p* Values and false discovery rates for the iTRAQ ratios were calculated using the ProteinPilot software. Proteins with iTRAQ ratios with *p* values ≤ 0.05 and with differential abundance of greater than or equal to ± 2.0-fold change (FC) were used in further analysis.

### Verification of iTRAQ nanoLC-MS/MS results using enzyme linked immunosorbent assay

Two proteins of biological relevance were measured by enzyme-linked immunosorbent assay (ELISA) in the non-pooled samples to verify the MS findings. Firstly, complement C1S subcomponent (C1s) was selected because this protein demonstrated differential abundance between responders and non-responders to ACI within the baseline SF (prior to stage I surgery) and therefore could have potential as a biomarker of outcome prediction. C1s was assessed using a human ELISA (CUSABIO, Houston, TX, USA). Samples were first assayed using a 1:100 dilution in assay sample diluent, and for those samples that were undetectable in the assay, the assay was repeated using undiluted samples. Secondly, matrix metalloproteinase 3 (MMP3) was selected to investigate the differential response to stage I surgery (i.e., the proteomic shift between stages I and II) in non-responders to ACI. MMP3 was assessed using a human Quantikine^®^ ELISA (R&D Systems, Abingdon, UK). Samples were diluted 1:100 in assay kit diluent prior to assessment. Both ELISAs were carried out according to the manufacturer’s instructions, and protein concentrations were normalised to the sample dilution factor. Statistical analysis was performed using Prism version 6.0 software (GraphPad Software, La Jolla, CA, USA). Student’s *t* tests were used to assess differential abundance.

### Assessment of protein overlap identified using the two proteomic approaches

To assess whether the use of two independent proteomic approaches allows for a greater number of significant protein changes to be identified, the datasets from this study (iTRAQ nanoLC-MS/MS [nLC-MS/MS]) and our previously published study assessing the same patient samples (LF LC-MS/MS [3]) were compared with one another. Venn diagrams were plotted using VENNY 2.1.0 software [37] to assess the overlap of differentially abundant proteins that were identified via the two approaches.

### Pathway and network analysis of proteomic datasets

The datasets generated using both proteomic approaches were combined. Specifically, proteins that were differentially expressed (≥ 1.2 FC; *p* ≤ 0.05) in each biological comparison (e.g., stage I responders versus non-responders) in either proteomic approach were merged into a single dataset. A modest FC cutoff was used to ensure that the greatest number of differentially abundant proteins could be included in the pathway and network analyses, as has been done previously [3, 18]. The iTRAQ nLC-MS/MS dataset independently, as well as when merged with the LF dataset, was analysed using pathway enrichment analysis (Ingenuity Pathway Analysis; Qiagen Bioinformatics, Redwood City, CA, US) to identify and visualise affected canonical pathways. Pathways with a significance level of *p* ≤ 0.005 were considered statistically significant (Fisher’s exact test).

The merged LF and iTRAQ nLC-MS/MS datasets of proteomic response to cartilage harvest (e.g., differential abundance between stages I and II) in responders and non-responders were assessed using interactome network analysis, which is an unbiased mathematical method of visualising and interpreting complex interactions between large numbers of molecules [38]. Interactome networks are made up of nodes (the individual objects being studied, such as proteins) and edges (the connections between the objects, such as known protein-protein interactions) [39]. By studying groups of proteins that are highly interconnected, known as *modules*, key functions within an interactome network can be highlighted [39]. Conducting interactome network analysis alongside pathway enrichment analysis allows for greater confidence in the selection of candidate pathways or molecules for further study, because these represent two independent methods of mapping the data: known protein-protein interactions and text mining, respectively. The interactions between the differentially abundant proteins were assessed using the PINA4MS (Protein Interaction Network Analysis For Multiple Sets) app [40] in Cytoscape version 3.0 to generate network models based on protein-protein interactions. These models were based either on only those proteins identified in the proteomic analyses (non-inferred nodes) or on proteins identified in the proteomic analyses alongside their inferred interactions (inferred nodes) [41]. The ModuLand (version 2.8.3) algorithm [42] was applied to the interactome networks in Cytoscape version 3.0 to identify highly connected clusters of proteins (modules) that demarcate the hierarchical structure of the interactome network. The biological function of each module was assessed by analysing the proteins identified within each module using the pathway analysis tool in Reactome software [43, 44]. The significance of the pathway functions identified in Reactome was determined by Fisher’s exact test, and *p* ≤ 0.05 was considered statistically significant.

## Results

The proteomic data derived from this study have been deposited in the PRoteomics IDEntifications (PRIDE) ProteomeXchange and can be accessed using the identifier [PXD008321].

### Identification of proteins to predict ACI outcome prior to stage I or stage II

iTRAQ nLC-MS/MS highlighted 16 proteins (greater than or equal to ± 2.0 FC; *p* ≤ 0.05) that were differentially abundant between responders and non-responders to ACI at baseline (immediately prior to stage I) (Table 2). Prior to stage II of the ACI procedure, 12 proteins displayed differential abundance between responders and non-responders (Table 3).

**Table 2:** Fold change of proteins that are differentially abundant in the synovial fluid of clinical non-responders compared with clinical responders to autologous chondrocyte implantation (ACI) immediately prior to stage I

Protein		Fold change	Identification method	
Description	Accession no.		LF LC-MS/MS	iTRAQ nLC-MS/MS
Complement C1S subcomponent	P09871	− 5.15		+
Haptoglobin	P00738	− 4.49		+
Mesencephalic astrocyte-derived neurotrophic factor	P55145	2.15		+
Plasma protease C1 inhibitor	P05155	2.19		+
Immunoglobulin kappa chain V-II region MIL	P01615	2.60	+	
Bifunctional glutamate/proline-transfer RNA ligase	P07814	2.61		+
Pigment epithelium-derived factor	P36955	3.13		+
Apolipoprotein A-IV	P06727	3.19		+
Apolipoprotein L1	O14791	3.19		+
*N*-acetylglucosamine-6-sulphatase	P15586	3.25		+
Retinol-binding protein 4	P02753	3.34		+
Inter-alpha-trypsin inhibitor heavy chain H1	P19827	3.37		+
Extracellular matrix protein 1	Q16610	3.77		+
Lumican	P51884	3.80		+
Histidine-rich glycoprotein	P04196	3.84		+
Endoplasmin	P14625	4.37		+
Serum paraoxonase/arylesterase 1	P27169	4.41		+

Footnote: Differential abundance was denoted by greater than or equal to ± 2.0-fold change; *p* ≤ 0.05; protein identified by at least two unique peptides.  Positive numbers denote higher abundance in non-responders than in responders. Proteins were identified using either protein dynamic compression coupled with label-free quantitation LC-MS/MS or no protein dynamic compression with isobaric tags for absolute and relative quantitation (iTRAQ) LC-MS/MS

**Table 3:** Fold change of proteins that are differentially abundant in the synovial fluid of clinical non-responders compared with clinical responders to autologous chondrocyte implantation (ACI) immediately prior to stage II

Protein		Fold Change	Identification method	
Description	Accession		LF LC-MS/MS	iTRAQ nLC-MS/MS
40S Ribosomal protein S14	P62263	− 8.63		+
Kinectin	Q86UP2	− 6.20		+
Apolipoprotein C-III	P02656	− 2.78		+
High-mobility group protein B1	P09429	− 2.56		+
Kininogen-1	P01042	2.27		+
26S Protease regulatory subunit 7	P35998	2.34	+	
26S Proteasome non-ATPase regulatory subunit 13	Q9UNM6	2.43	+	
Alpha-enolase	P06733	2.56		+
Alpha-2-HS-glycoprotein	P02765	2.78		+
Hemopexin	P02790	2.88		+
Ferritin light chain	P02792	2.91	+	
Platelet factor 4	P02776	3.26	+	
Thrombospondin-1	P07996	3.40	+	
Nucleosome assembly protein 1-like 1	P55209	4.94	+	
Cofilin-1	P23528	7.08	+	
EH domain-containing protein 1	Q9H4M9	7.30	+	
Haemoglobin subunit delta	P02042	8.09		+
Protein S100-A6	P06703	8.39		+
T-complex protein 1 subunit eta	Q99832	8.43	+	
Haemoglobin subunit beta	P68871	32.81		+
Haemoglobin subunit alpha	P69905	44.06		+

Footnote: Differential abundance was denoted by greater than or equal to ± 2.0-fold change; *p* ≤ 0.05; protein identified by at least two unique peptides. Positive numbers denote higher abundance in non-responders than in responders. Proteins were identified using either protein dynamic compression coupled with label-free quantitation LC-MS/MS or no protein dynamic compression with isobaric tags for absolute and relative quantitation (iTRAQ) LC-MS/MS

**Fig. 1:**
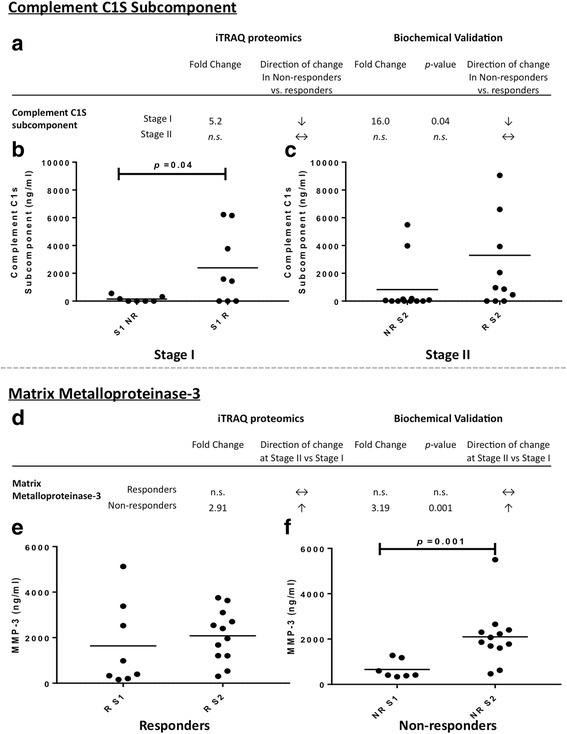
Biochemical validation of differentially abundant proteins identified using isobaric tagging for relative and absolute quantitation (iTRAQ) proteomics. **a** and **d** Differential abundance of complement C1S subcomponent (C1S) and matrix metalloproteinase 2 as measured by iTRAQ MS and biochemical enzyme-linked immunosorbent assay (ELISA), respectively. Quantitative ELISA confirmed that (**b**) C1S is significantly decreased in the synovial fluid (SF) of non-responders (NR) compared with responders (R) to autologous chondrocyte implantation (ACI) prior to cartilage harvest (stage I [S1]; *p* = 0.04 by Student’s *t* test) (**c**) but was not significantly differentially abundant prior to chondrocyte implantation (stage II [S2]). Matrix metalloproteinase 3 (MMP3) (**e**) was not differentially abundant in response to cartilage harvest in ACI responders (**f**) but was biochemically confirmed to be differentially abundant in the SF of non-responders between stages I and II of the ACI procedure (*p* = 0.001 by Student’s *t* test)

At both stages of treatment, SF analysed using iTRAQ nLC-MS/MS identified a greater number of differentially abundant proteins between individuals who did or did not respond well to ACI compared with SF that had undergone protein normalisation using the ProteoMiner™ protein enrichment kit (Bio-Rad Laboratories, Hercules, CA, USA) and LF LC-MS/MS analysis [3]. Further, the two proteomic techniques identified no common differentially abundant proteins. The two proteins selected and assessed by ELISA (C1s and MMP3) could verify the iTRAQ nLC-MS/MS (Fig. 1).

### Differential abundance of proteins at stage II compared with stage I of ACI

**Fig. 2:**
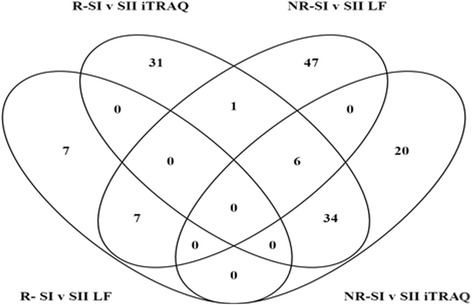
Venn diagrams representing the proteins identified using isobaric tags for relative and absolute quantitation (iTRAQ) proteomics and label-free quantitation (LF) proteomics. The proteins shown were differentially abundant (≥ 2.0-fold change; *p* ≤ 0.05) in the SF at stage I (SI) compared with stage II (SII) in responders (R) compared with non-responders (NR) to ACI

Proteomic profiling of the SF using iTRAQ nLC-MS/MS highlighted a considerable effect of the cartilage harvest procedure (stage I) in both responders and non-responders, with 70 and 54 proteins being differentially abundant between stages I and II, respectively, thus strengthening the similar findings derived from the analysis of these samples using LF LC-MS/MS [3]. Interestingly, the iTRAQ nLC-MS/MS and LF LC-MS/MS identified no common protein differences between stage I and stage II in the clinical responders (70 differentially abundant proteins identified by iTRAQ nLC-MS/MS and 14 identified by LF LC-MS/MS) (Table 4). This lack of overlap between the two proteomic techniques is highlighted in Fig. 2. There were, however, six proteins (gelsolin, vitamin K-dependent protein S, C4b-binding protein alpha chain, fibrinogen alpha chain, fibrinogen beta chain and fibrinogen gamma chain) that were identified by both proteomic techniques in the non-responders, all of which showed commonality in the direction of protein shift across the MS platforms, with iTRAQ nLC-MS/MS consistently resulting in greater differences in abundance than those identified from the LF LC-MS/MS data. A total of 54 protein abundance changes between stages I and II in non-responders were identified using iTRAQ nLC-MS/MS, and 55 protein differences were identified by LF LC-MS/MS (Table 5 and Fig. 2).

**Table 4:** Fold change of proteins that are differentially abundant in the synovial fluid of clinical responders at stage II compared with stage I of autologous chondrocyte implantation (ACI)

Protein		Fold change	Identification method	
Description	Accession no.		LF LC-MS/MS	iTRAQ nLC-MS/MS
Microtubule-associated protein 1B	P46821	− 20.65	+	
40S Ribosomal protein S14	P62263	− 16.75		+
Protein disulphide-isomerase A6	Q15084	− 7.59		+
Nucleolin	P19338	− 5.11		+
Histone H1.2	P16403	− 3.84		+
Stress-induced-phosphoprotein 1	P31948	− 3.63		+
Complement factor D	P00746	− 3.44		+
SH3 domain-binding glutamic acid-rich-like protein	O75368	− 3.44		+
Heterogeneous nuclear ribonucleoprotein U	Q00839	− 3.40		+
78 kDa Glucose-regulated protein	P11021	− 3.25		+
Cartilage oligomeric matrix protein	P49747	− 3.10		+
Annexin A2	P07335	− 2.96		+
Mesencephalic astrocyte-derived neurotrophic factor	P55145	− 2.86		+
Kinectin	Q86UP2	− 2.81		+
Complement factor H-related protein 3	Q02985	− 2.77	+	
Phosphatidylethanolamine-binding protein 1	P30086	− 2.51		+
Peroxiredoxin-4	Q13162	− 2.49	+	
Regucalcin	Q15493	− 2.44		+
Malate dehydrogenase, mitochondrial	P40926	− 2.44		+
*N*-acetylglucosamine-6-sulphatase	P15586	− 2.31		+
Gelsolin	P06396	− 2.27		+
Alpha-endosulfine	O43768	− 2.25		+
Peptidyl-prolyl cis-trans isomerase FKBP3	Q00688	− 2.11		+
Hemopexin	P02790	2.05		+
Serum paraoxonase/arylesterase 1	P27169	2.07		+
Secreted phosphoprotein 24	Q13103	2.10	+	
Heparin cofactor 2	P05546	2.13		+
Ferritin light chain	P02792	2.21	+	
Attractin	O75882	2.21		+
Ig gamma-2 chain C region	P01859	2.23		+
Plasma kallikrein	P03952	2.24		+
Chondroitin sulphate proteoglycan 4	Q6UVK1	2.35	+	
Collagen alpha-2(I) chain	P08123	2.37	+	
Collagen alpha-1(V) chain	P20908	2.54	+	
CD5 antigen-like	O43866	2.58		+
Phospholipid transfer protein	P55058	2.63		+
Insulin-like growth factor-binding protein complex acid labile subunit	P35858	2.68		+
Prothrombin	P00734	2.68		+
Beta-2-glycoprotein 1	P02749	2.78		+
Collagen alpha-2(V) chain	P05997	2.84	+	
Plasma protease C1 inhibitor	P05155	2.91		+
Serum amyloid P component	P02743	2.91		+
Complement C1q subcomponent subunit B	P02746	3.01		+
Collagen alpha-1(I) chain	P02452	3.05	+	
Alpha-2-antiplasmin	P08697	3.10		+
Alpha-1B-glycoprotein	P04217	3.19		+
Complement factor B	P00751	3.25		+
Complement component C7	P10643	3.40		+
Vitamin K-dependent protein S	P07225	3.42		+
Apolipoprotein E	P02649	3.44		+
Alpha-1-antichymotrypsin	P01011	3.44		+
Carboxypeptidase N subunit 2	P22792	3.53		+
Vitronectin	P04004	3.63		+
Inter-alpha-trypsin inhibitor heavy chain H3	Q06033	3.66		+
Complement C5 O	P01031	4.00		+
Plasminogen	P00747	4.06		+
Kininogen 1	P01042	4.17		+
Platelet factor 4	P02776	4.26	+	
Inter-alpha-trypsin inhibitor heavy chain H2	P19823	4.49		+
Periostin	Q15063	4.57	+	
Apolipoprotein L1	O14791	4.61		+
Protein 4.1	P11171	4.66	+	
26S Proteasome non-ATPase regulatory subunit 13	Q9UNM6	4.78	+	
Inter-alpha-trypsin inhibitor heavy chain H1	P19827	5.01		+
Inter-alpha-trypsin inhibitor heavy chain H4	Q14624	5.06		+
Complement C1r subcomponent	P00736	5.15		+
Complement component C6	P13671	5.45		+
Complement factor H	P08603	5.50		+
Catalase	P04040	5.60		+
Ficolin-3	O75636	6.43		+
C4b-binding protein alpha chain	P04003	7.05		+
Ceruloplasmin	P00450	7.51		+
Pregnancy zone protein	P20742	8.09		+
Fibrinogen alpha chain	P02671	8.40		+
Apolipoprotein M	O95445	9.04		+
Protein S100-A6	P06703	9.82		+
Haemoglobin subunit alpha	P69905	9.82		+
Complement C1s subcomponent	P09871	10.00		+
Ig mu chain C region	P01871	12.13		+
Haptoglobin	P00738	13.68		+
Fibrinogen beta chain	P02675	16.90		+
Haemoglobin subunit beta	P68871	19.41		+
Fibrinogen gamma chain	P02679	23.55		+

Footnote: Differential abundance was denoted by greater than or equal to ± 2.0-fold change; *p* ≤ 0.05; protein identified by at least two unique peptides. Positive numbers denote higher abundance at stage II than at stage I. Proteins were identified using either protein dynamic compression coupled with label-free quantitation LC-MS/MS or no protein dynamic compression with isobaric tags for absolute and relative quantitation (iTRAQ) LC-MS/MS

**Table 5:** Fold change of proteins that are differentially abundant in the synovial fluid of clinical non-responders at stage II compared with stage I

Protein		Fold change	Identification method	
Description	Accession		LF LC-MS/MS	iTRAQ nLC-MS/MS
Protein S100-A6	P06703	− 4.49		+
Annexin A1	P04083	− 4.13		+
Haemoglobin subunit beta	P68871	− 4.09		+
Complement factor D	P00746	− 3.87		+
Perilipin-4	Q96Q06	− 3.87	+	
*Gelsolin*	*P06396*	− 3.31		+
*Gelsolin*	*P06396*	− 1.68	+	
Syntaxin 7	O15400	− 3.31	+	
Fermitin family homolog 3	Q86UX7	− 3.29	+	
Histone H1.2	P16403	− 3.13		+
Transaldolase	P37837	− 3.08		+
Neuroblast differentiation-associated protein AHNAK	Q09666	− 2.78	+	
Heterogeneous nuclear ribonucleoprotein K	P61978	− 2.69	+	
Hyaluronan and proteoglycan link protein 3	Q96S86	− 2.65	+	
Alpha-enolase	P06733	− 2.63		+
ATP-citrate synthase	P53396	− 2.63	+	
Annexin A2	P07355	− 2.56		+
Fatty acid-binding protein, epidermal	Q01469	− 2.43	+	
Peroxiredoxin-1	Q06830	− 2.20	+	
Tripeptidyl-peptidase 1	O14773	− 2.19	+	
Insulin-like growth factor-binding protein 6	P24592	− 2.13	+	
Na^+^/H^+^ exchange regulatory cofactor NHE-RF1	O14745	− 2.11	+	
Peroxiredoxin-6	P30041	− 2.08	+	
Histamine N-methyltransferase	P50135	− 2.07	+	
Mortality factor 4-like protein 1	Q9UBU8	− 2.06	+	
Transcription elongation factor A protein 1	P23193	− 2.06	+	
Cartilage acidic protein 1	Q9NQ79	− 2.03		+
2′,3′-cyclic-nucleotide 3′-phosphodiesterase	P09543	− 1.20	+	
Fructose-bisphosphate aldolase A	P04075	− 1.97	+	
Leucine zipper transcription factor-like protein 1	Q9NQ48	− 1.94	+	
Protein S100-A13	Q99584	− 1.94	+	
40S Ribosomal protein S3	P23396	− 1.93	+	
Filamin-A	P21333	− 1.92	+	
Microtubule-associated protein RP/EB family member 1	Q15691	− 1.92	+	
Nuclear migration protein nudC	Q9Y266	− 1.90	+	
Prostaglandin E synthase 3	Q15185	− 1.85	+	
Stress-induced phosphoprotein 1	P31948	− 1.85	+	
Cytokine-like protein 1	Q9NRR1	− 1.81	+	
Plastin-2	P13796	− 1.81	+	
Coronin-1C	Q9ULV4	− 1.80	+	
Vinculin	P18206	− 1.80	+	
Cathepsin K	P43235	− 1.79	+	
Hsc70-interacting protein	P50502;Q8IZP2	− 1.76	+	
Putative phospholipase B-like 2	Q8NHP8	− 1.74	+	
Spectrin beta chain, erythrocytic	P11277	− 1.73	+	
Complement factor I	P05156	2.11		+
Alpha-1-antichymotrypsin	P01011	2.22		+
Titin	Q8WZ42	2.23		+
Cytoplasmic dynein 1 heavy chain 1	Q14204	2.23	+	
F-actin-capping protein subunit beta	P47756	2.25	+	
Mannan-binding lectin serine protease 1	P48740	2.26	+	
Serum amyloid P-component	P02743	2.27		+
Complement component C6	P13671	2.29		+
Thrombospondin-3	P49746	2.36	+	
Soluble scavenger receptor cysteine-rich domain-containing protein SSC5D	A1L4H1	2.39	+	
Plasma kallikrein	P03952	2.42		+
Complement factor B	P00751	2.47		+
Afamin	P43652	2.47		+
*Vitamin K-dependent protein S*	P07225	2.49	+	
*Vitamin K-dependent protein S*	P07225	3.08		+
Integrin beta-like protein 1	O95965	2.51	+	
C4b-binding protein beta chain	P20851	2.55	+	
Fibronectin	P02751	2.58	+	
Clusterin	P10909	2.65		+
Vitronectin	P04004	2.68		+
Bifunctional glutamate/proline-transfer RNA ligase	P07814	2.70		+
Nucleobindin 1	Q02818	2.71	+	
Complement component C9	P02748	2.75		+
Zinc-alpha-2-glycoprotein	P25311	2.75		+
Complement C1r subcomponent	P00736	2.83		+
Heparin cofactor 2	P05546	2.83		+
Ferritin light chain	P02792	2.84	+	
Proteoglycan 4	Q92954	2.88		+
*C4b-binding protein alpha chain*	P04003	2.91	+	
*C4b-binding protein alpha chain*	P04003	10.38		+
*Matrix metalloproteinase 3*	P08254	2.91		+
Attractin	O75882	2.94		+
Insulin-like growth factor-binding protein complex acid labile subunit	P35858	3.02		+
Alpha-1B-glycoprotein	P04217	3.05		+
*Fibrinogen alpha chain*	P02671	3.10	+	
*Fibrinogen alpha chain*	P02671	11.91		+
Lumican	P51884	3.13		+
Chondroitin sulphate proteoglycan 4	Q6UVK1	3.16	+	
Collagen alpha-2(V) chain	P05997	3.19	+	
Complement C2	P06681	3.22		+
*Fibrinogen beta chain*	P02675	3.25	+	
*Fibrinogen beta chain*	P02675	18.37		+
Secreted phosphoprotein 24	Q13103	3.26	+	
Matrix metalloproteinase 1	P03956	3.33	+	
Latent-transforming growth factor beta-binding protein 1	Q14766	3.45	+	
Phospholipid transfer protein	P55058	3.47		+
Inter-alpha-trypsin inhibitor heavy chain H3	Q06033	3.47		+
Complement C1q tumour necrosis factor-related protein 3	Q9BXJ4	3.50	+	
Adipocyte enhancer-binding protein 1	Q8IUX7;Q8N436	3.51	+	
Adiponectin	Q15848	3.52	+	
*Fibrinogen gamma chain*	P02679	3.79	+	
*Fibrinogen gamma chain*	P02679	18.37		+
Plasminogen	P00747	3.84		+
Apolipoprotein C-II	P02655	3.94		+
CD5 antigen-like	O43866	4.17		+
Collagen alpha-1(V) chain	P20908	4.26	+	
Complement factor H	P08603	4.57		+
Inter-alpha-trypsin inhibitor heavy chain H4	Q14624	4.61		+
Complement C5	P01031	4.74		+
Collagen alpha-1(I) chain	P02452	4.84	+	
Ceruloplasmin	P00450	5.01		+
Histidine-rich glycoprotein	P04196	5.11		+
Target of Nesh-SH3	Q7Z7G0	5.25		+
Plasma protease C1 inhibitor	P05155	5.30		+
Apolipoprotein M	O95445	5.65		+
Inter-alpha-trypsin inhibitor heavy chain H2	P19823	5.70		+
Periostin	Q15063	5.81	+	
Apolipoprotein C-III	P02656	5.92		+
Kinectin	Q86UP2	6.02		+
Carboxypeptidase N subunit 2	P22792	7.73		+
Serum paraoxonase/arylesterase 1	P27169	8.02		+
Apolipoprotein L1	O14791	8.95		+
Ig mu chain C region	P01871	13.30		+
Apolipoprotein E	P02649	13.80		+
Inter-alpha-trypsin inhibitor heavy chain H1	P19827	15.56		+

Footnote: Differential abundance was denoted by greater than or equal to ± 2.0-fold change; *p* ≤ 0.05; protein identified by at least two unique peptides. Positive numbers denote higher abundance at stage II compared with stage I of autologous chondrocyte implantation (ACI). Proteins were identified using either protein dynamic compression coupled with label free quantitation LC-MS/MS or no protein dynamic compression with isobaric tags for absolute and relative quantitation (iTRAQ) LC-MS/MS. Proteins identified by both proteomic techniques are underlined and in italics

### iTRAQ nLC-MS/MS confirmed a significant response to cartilage harvest procedure (stage I) in nonresponders to ACI

Pathway analysis of the iTRAQ nLC-MS/MS-identified proteins, using the pathway enrichment tools in Ingenuity Pathway Analysis, suggested that the proteins which were differentially abundant at stage II compared with stage I in non-responders are likely to impact numerous canonical pathways, many of which were confirmatory of the previously published functional pathways identified from the LF nLC-MS/MS-derived proteins [3]. These functional pathways included acute-phase response signalling (*p* = 2.93 × 10^− 1^), the complement system (*p* = 2.11 × 10^− 1^) and liver X receptor/retinoic X receptor signalling (*p* = 1.95 × 10^− 1^). Moreover, many more functional pathways were affected as a result of the proteins that were differentially abundant in response to stage II compared with stage I in non-responders compared with responders (Additional file 1: Tables S1 and S2), reiterating that the SF proteomic response to cartilage harvest is more distinct in non-responders to ACI.

### Similar pathways were identified from the differentially abundant proteins identified in iTRAQ nLC-MS/MS and LF LC-MS/MS analyses

**Fig. 3:**
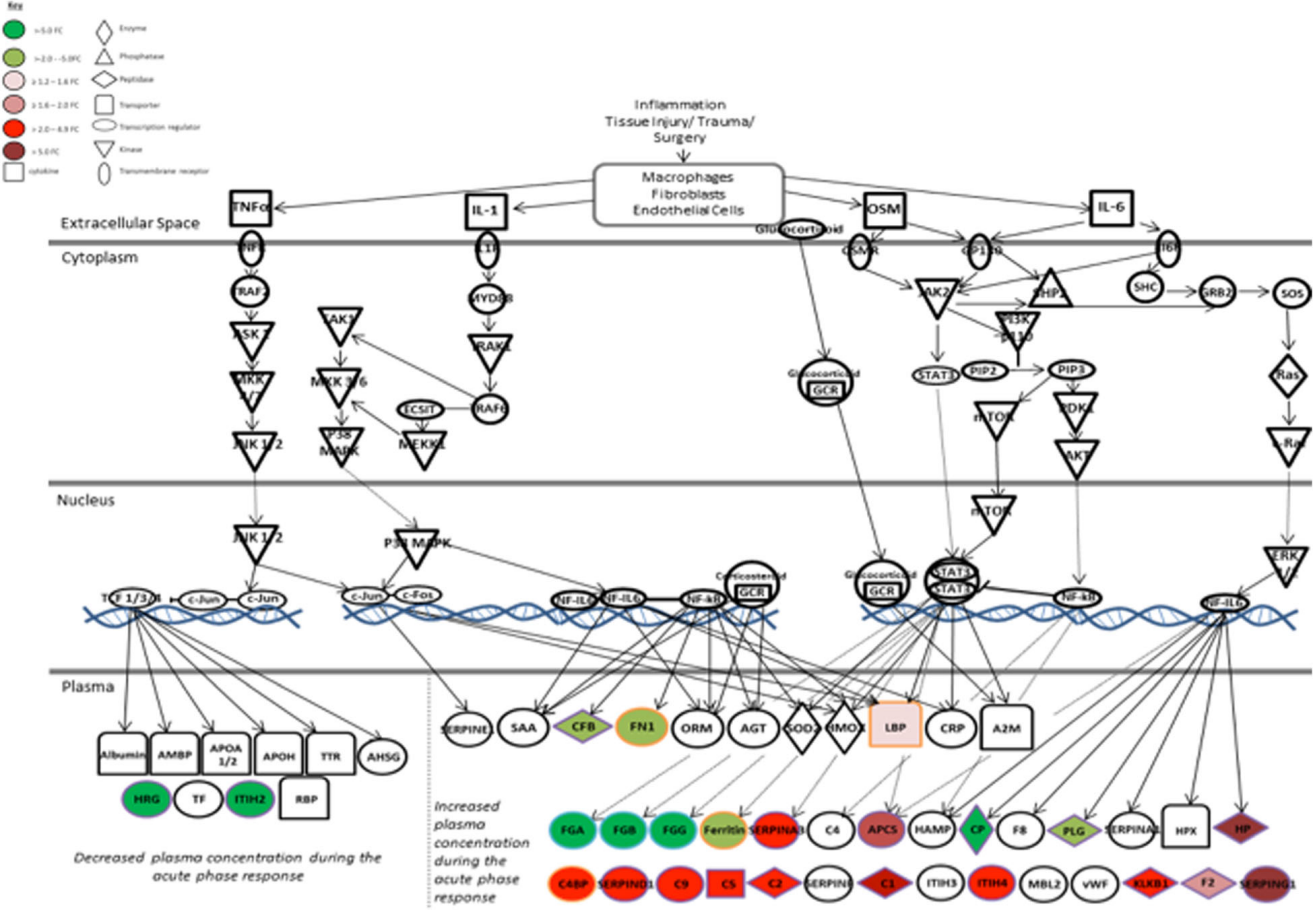
Proteins of acute-phase signalling at stage II compared with stage I in non-responders to autologous chondrocyte implantation (ACI). Several synovial fluid proteins that are downstream of acute-phase response signalling were differentially abundant between stages I and II of ACI. Proteins edged in *purple*, *orange* and *blue* were identified using isobaric tagging for relative and absolute quantitation (iTRAQ) nano LC-MS/MS, label-free quantitation (LF) LC-MS/MS or both techniques, respectively. (*Adapted from Ingenuity Pathway Analysis.*)

Both iTRAQ nLC-MS/MS and LF LC-MS/MS analyses resulted in acute-phase response signalling being highlighted as one of the most significantly affected pathways in response to cartilage harvest in non-responders to ACI; therefore, this pathway was further assessed. Figure 3 highlights that analysis of the SF proteome using the two independent proteomic techniques resulted in a greater number of differentially abundant downstream proteins being identified. In addition, many complementary proteins have been identified when comparing these datasets, with the vast majority of proteins that are predicted to be increased in the plasma (the standard bodily fluid referred to in Ingenuity Pathway Analysis) during the acute-phase response being more abundant in the SF at stage II than at stage I and vice versa.

Because the results of the two proteomic approaches seem to be complementary to one another, the two datasets were combined to generate a more comprehensive profile of the SF proteome. Ingenuity Pathway Analysis again identified many functional pathways similar to those identified via the independent LF LC-MS/MS and iTRAQ nLC-MS/MS datasets. The most significant canonical pathways associated with the non-responder response to cartilage harvest (stage II versus stage I) were acute-phase response signalling (*p* = 1.10 × 10^− 9^), intrinsic prothrombin activation pathway (*p* = 3.43X10^− 7^) and the complement system (*p* = 1.22 × 10^− 6^). Further, analysis of upstream regulators to these dysregulated proteins included those identified using the LF LC-MS/MS analysis data alone, such as transforming growth factor-β1 (*p* = 2.05 × 10^− 13^), dihydrotestosterone (*p* = 4.48 × 10^− 11^) and peroxisome proliferator-activated receptor-α (*p* = 1.09 × 10^− 9^) [3].

**Fig. 4:**
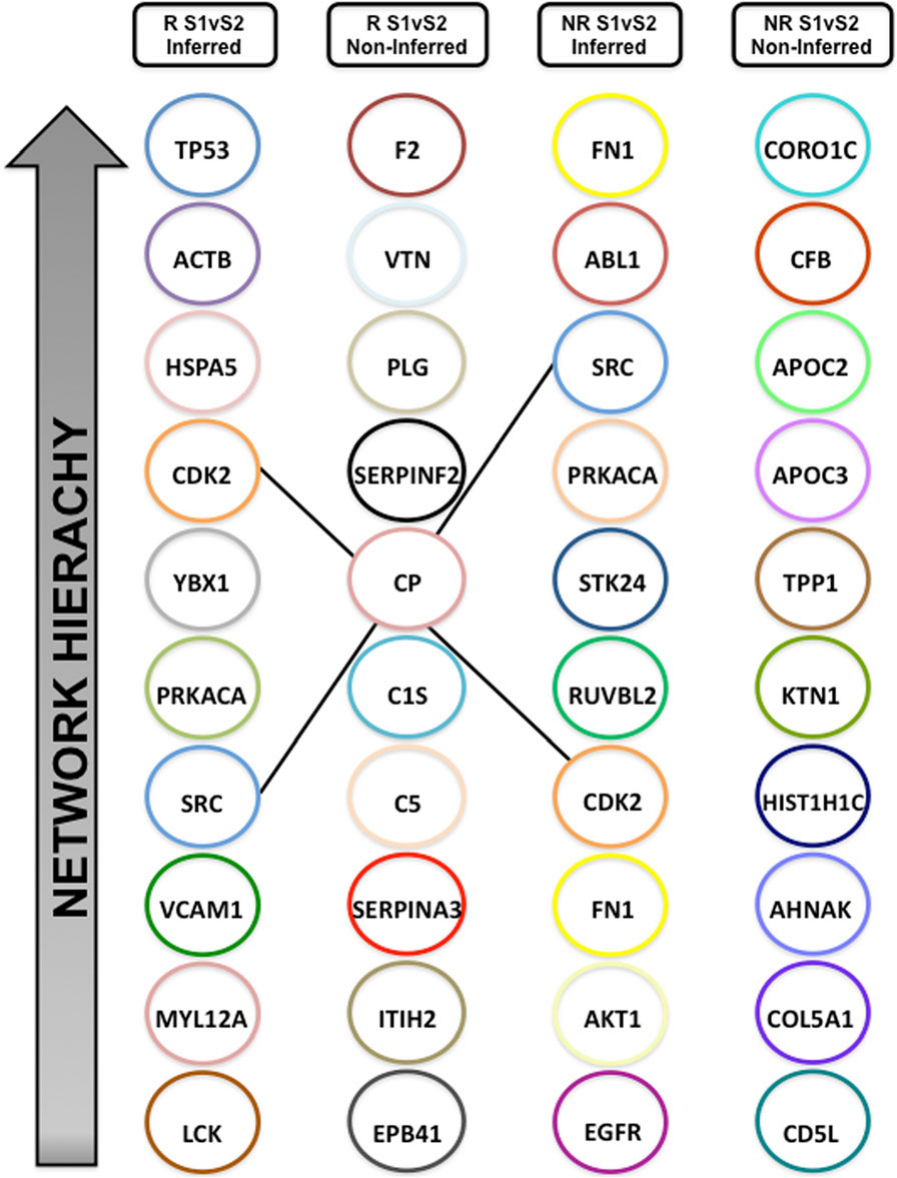
The ModuLand algorithm was applied in Cytoscape to inferred and non-inferred interactome networks of differentially abundant proteins (± 1.2-fold change; *p* ≤ 0.05) between stages I and II of autologous chondrocyte implantation in clinical responders and non-responders. Modules were identified from both non-inferred (protein changes identified from proteomic analysis only) and inferred (identified protein changes and inferred proteins interactions) networks and are ranked on the basis of their hierarchical network connectivity

The combined datasets were then used to generate unbiased interactome networks that represent the differentially abundant proteins (non-inferred networks), their likely interacting proteins (inferred networks) and how these proteins interact with one another, resulting in models of systemic protein response to cartilage harvest in either the responders or non-responders to ACI. Based on proteins that were differentially abundant between stages I and II of ACI in non-responders, an interactome network consisting of 115 nodes (proteins) and 40 edges (protein-protein interactions) was generated. Further, an inferred network consisting of 2893 proteins and 35,576 protein-protein interactions was generated on the basis of the addition of proteins that are likely to interact with the differentially abundant proteins (PINA4MS interactome database). Proteins that were differentially abundant in response to cartilage harvest in responders to ACI were used to generate interactome networks (non-inferred, 83 nodes and 118 edges; inferred, 2084 nodes and 54,007 edges). The ModuLand algorithm was applied to each of these networks to identify modules within the network that can be hierarchically ranked to identify groups of proteins that are the most fundamental in the functioning of the network. Figure 4 highlights the top ten modules from each of the networks generated. These modules again highlight the disparity between the ACI responder and non-responder response to cartilage harvest, with only modules centred on the proto-oncogene tyrosine-protein kinase (Src) protein being identified in the inferred networks of both non-responder and responder groups. Interestingly, assessment of the functional pathways related to the ModuLand identified modules in the non-responder networks again highlighted regulation of the complement cascade (*p* = 1.68 × 10^− 8^ by Fisher’s exact test), thus providing confidence in its importance based on identification via two independent bioinformatics approaches.

## Discussion

On the basis of its recent technology appraisal of ACI, NICE has recommended this treatment for a specific subset of patients with cartilage injury in the knee [2]. The identification of novel biomarkers that can strengthen current patient demographic risk factors in predicting clinical outcomes [9], as well as development of a greater understanding of the underlying biology associated with success and failure, will be beneficial, particularly because this treatment option is likely to be implemented on a wider scale in the near future. The present study builds upon our previously published work [3, 8], highlighting a number of novel protein candidates that have potential as biomarkers to predict ACI outcome. Moreover, comprehensive proteomic profiling of SF has further highlighted proteomic differences between responders and non-responders to ACI.

In the majority of studies in which the SF proteome has been profiled, either high-abundance proteins [11–16] or low-abundance proteins [17–22] have been assessed via depletion or non-depletion of abundant proteins prior to proteomic analysis. Our study highlights that the use of both a proteomic dynamic range compression technique (ProteoMiner™) [3] in tandem with analysis of non-depleted SF samples can provide a more holistic overview of proteomic changes, because both iTRAQ nLC-MS/MS and LF LC-MS/MS highlighted large numbers of differentially abundant proteins between stages I and II of ACI, with little crossover between techniques. This type of all-inclusive approach to unbiased whole-proteome analysis of biological fluids may therefore be more successful in the identification of candidate biomarkers for treatments/disease states beyond those we investigated.

A limitation of our previous study [3] was that very few proteins were identified as differentially abundant between responders and non-responders at baseline. In order for biomarkers aimed at predicting ACI success to be most clinically useful, patients who are likely to fail or respond to this procedure need to be identified prior to any surgical intervention. Interestingly, analysis of non-dynamic range compressed proteins with iTRAQ nLC-MS/MS analysis was able to detect a greater number of differentially abundant proteins between responders and non-responders prior to stage I surgery. The protein with most altered abundance in responders compared with non-responders at stage I was C1s. This higher abundance in responders was confirmed in individual patient samples using a biochemical assay. C1s is a major constituent of the trimeric complement C1 protein, which triggers the classical complement pathway. Once activated, the classical complement pathway promotes inflammation to enable the removal of damaged cells and/or microbes. Moreover, C1s has been shown to cleave insulin-like growth factor 1 (IGF-1) [45] and insulin-like growth factor binding protein 5 (IGFBP-5) [46]. Both IGF-1 and IGFBP-5 are chondroprotective when in their intact state [45, 47], and inhibition of C1s activity within the canine SF reduced cleavage of IGFBP-5 and IGF-1, resulting in reduced cartilage damage following anterior cruciate ligament rupture [45]. These studies indicate that high C1s activity levels are likely detrimental to cartilage repair. Further, the complement cascade is known to be important in the pathogenesis of OA, with patients with OA demonstrating increased gene expression of complement agonists compared  to inhibitors [48]. OA-related pathogenesis, such as the release of cartilage extracellular matrix molecules and the production of inflammatory mediators, induces complement activation [48]. The increased pre-operative levels that we identified in individuals who responded well to ACI perhaps indicate that ACI has potential to be successful in individuals who may have developed an early OA phenotype.

Analysis of the iTRAQ nLC-MS/MS and LF LC-MS/MS datasets, both independently and when combined, highlighted that there is a marked proteomic shift in response to cartilage harvest (i.e., between stages I and II of ACI). This analysis resulted in a plethora of candidate biomarkers that may have the potential to be informative regarding whether an individual is likely to respond well to ACI prior to chondrocytes being implanted during stage II. The proteoglycan, collagens II-, IX- and X-degrading enzyme, MMP3 [49] has been biochemically validated as one of these candidate proteins that is significantly increased at stage II compared with stage I only in non-responders to ACI. Use of these biomarkers could have the potential to prevent the burden of a second surgery in a patient for whom this therapy is likely to be unsuccessful and could indicate that a greater period of time should be left from when the cartilage harvest procedure takes place to when the cells are implanted or that a tailored cartilage implantation procedure would be more efficacious.

To investigate the significant proteome shift that exists in response to cartilage harvest, pathway analyses were performed to better distinguish the underlying biological mechanisms that dictate whether an individual will respond to ACI. The acute-phase response was the pathway predicted to be most significantly differentially regulated in response to cartilage harvest in non-responders to ACI. In-depth assessment of individual protein changes within this pathway again highlighted the benefit of using independent proteomic techniques to profile the SF, because a large number of proteins were differentially abundant between stages I and II, only three of which were identified using both techniques. The acute-phase response is the body’s first systemic response to immunological stress, trauma and surgery [50]. At the site of injury/trauma, pro-inflammatory cytokines are normally released, activating inflammatory cells and ultimately resulting in inflammatory mediators and cytokines being released into the extracellular fluid compartment to be circulated in the blood [50]. Interestingly, previous bioinformatics analyses of the proteome of patients with late OA compared with healthy control subjects highlighted a dysregulated acute-phase response in the end-stage OA cohort [18]. The exacerbated activation of the acute-phase response in non-responders following initial surgery could indicate that these patients have a greater immune response to surgery and that they have a lesser ability to dampen the acute-phase following surgery or that they have already developed an advanced OA phenotype, deeming a therapy to repair cartilage injury unsuitable.

Finally, the datasets of combined iTRAQ nLC-MS/MS and LF LC-MS/MS identified proteins were used to generate interactome models that represent the systemic proteomic response to cartilage harvest which exists within the SF of both ACI responders and non-responders, from which biological functional pathways could be further studied. Biological functional pathways that were identified using this approach, as well as using Ingenuity Pathway Analysis can most confidently be taken forward as candidates for further study because they have been identified by independent bioinformatic methods. Furthermore, given the complexity of the knee joint environment, it is likely that the responder/non-responder phenotype is the result of many subtle protein changes which together contribute to overall dysfunction of a biological network, rather than being the result of an individual biological molecule or pathway per se. Therefore, the interactome models generated in this study provide an important opportunity to consider how these proteins interact with one another and result in such phenotypes, and they also provide a platform for further studies to investigate how potential modifications to the ACI procedure (e.g., using co-incidental anti-inflammatory drugs in non-responders at stage II) may alter these biological networks. Thus, these models may provide a potential *in silico *tool for predicting ACI outcome, as is commonly used in drug development strategies [51].

## Conclusions

This study highlights the advantage of using two independent proteomic techniques to profile a holistic overview of the SF proteome, ideal for unbiased identification of biomarker candidates. iTRAQ nLC-MS/MS analysis of SF samples from individuals who have either responded well or very poorly to ACI has highlighted proteins that, with further validation, have the potential to predict clinical outcome prior to treatment. We have confirmed that there is a marked SF proteome shift following cartilage injury, which is exacerbated in non-responders. Network and pathway analyses have demonstrated the complexity of the biological response underlying this proteome shift in non-responders, with several biological pathways identified that may act as targets for therapeutic intervention.

## Additional file

Additional file 1: **Table S1.** Canonical pathways altered in the synovial fluid of clinical nonresponders at stage I compared with stage II of ACI, identified using Ingenuity Pathway Analysis based on proteins that were identified using iTRAQ proteomics (≥ 1.2-fold change). Significance was assessed using a right-sided Fisher’s exact test; therefore, the most significant canonical pathways represent those that are the least likely to have been identified because of molecules being in the canonical pathway by random chance. The z-score represents canonical pathways that are likely activated or inhibited (based on the pattern of differentially abundant proteins); NaN means no prediction could be made based on the number of differentially abundant proteins in the pathway. **Table S2.** Canonical pathways altered in the synovial fluid of clinical responders at stage I compared with stage II of ACI, identified using Ingenuity Pathway Analysis, based on proteins which were identified using iTRAQ proteomics (≥ 1.2-fold change). Significance was assessed using a right-sided Fisher’s exact test; therefore, the most significant canonical pathways represent those that are the least likely to have been identified because of molecules being in the canonical pathway by random chance. The z-score represents canonical pathways that are likely activated or inhibited (based on the pattern of differentially abundant proteins); NaN means no prediction could be made based on the number of differentially abundant proteins in the pathway. (XLSX 19 kb)

## Abbreviations

ACI: Autologous chondrocyte implantation
BMI: Body mass index
C1s: Complement C1S subcomponent
ELISA: Enzyme-linked immunosorbent assay
FC: Fold change
IGF-1: Insulin-like growth factor 1
IGFBP-5: Insulin-like growth factor-binding protein 5
iTRAQ: Isobaric tagging for relative and absolute quantitation
LF: Label-free quantitation
LFC: Lateral femoral condyle
LTP: Lateral tibial plateau
MFC: Medial femoral condyle
MMP3: Matrix metalloproteinase 3
NICE: National Institute for Health and Care Excellence
nLC-MS/MS: Nano LC-MS/MS
OA: Osteoarthritis
PINA4MS: Protein Interaction Network Analysis For Multiple Sets
SCX: Strong cation exchange chromatography
SF: Synovial fluid
Src: Proto-oncogene tyrosine-protein kinase protein
